# Supplementary material for: Improved Applicability and Diagnostic Accuracy of the Novel Spleen-Dedicated Transient Elastography Device for High-Risk Esophageal Varices
Source: Diagnostics (Basel). 2024 Mar 30;14(7):743. doi: 10.3390/diagnostics14070743 (PMC11011917; doi:10.3390/diagnostics14070743)
Supplement: Supplementary file 1 [file diagnostics-14-00743-s001.zip › diagnostics-2932221-supplementary.pdf]

Supplementary table 1. Univariate and multivariate logistic regression model assessing independent predictors associated with the SSM> 40.9 kPa (as the indicator of presence of critical varices) in our cohort of patient (N=112). **Abbreviations:** ALBI: albumin-bilirubin score; ALT: alanine aminotransferase; AST: aspartate aminotransferase; APRI: AST to Platelet Ratio Index**;** BMI: body mass index; CAP: continuous attenuation parameter; dB/m: decibels per meter; CI: confidence interval; ECOG: Eastern Cooperative Oncology Group; FIB-4: Fibrosis-4; GGT: gamma glutamyl transferase; g/L: grams per liter; INR: international normalised ratio; IQR: interquartile range; kPa: kilopascal; LSM: liver stiffness measurement; M: median; MELD: model of end-stage liver disease; µmol/L: micromoles per liter; mmol/L: millimoles per liter; N: number; MASLD: Metabolic dysfunction-associated steatotic liver disease; PBC: primary biliary cholangitis; SSM: spleen stiffness measurement; U/L: units per liter

**Dependent variable: SSM>40.9 kPa**	**Odds ratio (univariate)**	**95% CI**
**Independent variables**		
**Age, years, median [IQR]**	1.012	0.979-1.046
**Age at time of diagnosis, years, median [IQR]**	1.005	0.968-1.044
**Male sex**	**3.312**	**1.329-8.251**
**BMI, kg/m ^{2}, median [IQR]**	1.040	0.956-1.132
**ECOG**<br>**0**<br>**1**<br>**2**	 Ref.<br>3.096<br>0.885	 <br>0.612-15.658<br>0.054-14.549
**Obesity (BMI>30 kg/m ^{2})**	1.731	0.745-4.032
**Arterial hypertension**	0.759	0.350-1.645
**Hyperlipidemia**	0.757	0.325-1.755
**Diabetes type II**	1.128	0.522-2.437
**Charlson Comorbidity Index**	1.667	0.982-1.386
**Etiology- Alcohol**	1.318	0.620-2.803
**Etiology- MASLD**	0.840	0.344-2.049
**Esophageal varices**	**16.260**	**6.137-43.084**
**Critical varices**	**26.400**	**3.407-204.562**
**Leukocytes, G/L, median [IQR]**	**0.756**	**0.625-0.914**
**Neutrophils, G/L, median [IQR]**	**0.749**	**0.590-0.950**
**Lymphocytes, G/L, median [IQR]**	**0.286**	**0.147-0.568**

**Neutrophile to lymphocyte ratio, median [IQR]**	1.150	0.872-1.516
**Hemoglobin, g/L median [IQR]**	0.993	0.976-1.011
**Platelets, G/L, median [IQR]**	**0.982**	**0.974-0.990**
**INR, median [IQR]**	**7.494**	**1.252-44.856**
**Bilirubin, µmol/L, median [IQR]**	1.008	0.989-1.026
**Creatinine, µmol/L, median [IQR]**	1.001	0.983-1.019
**Sodium, U/L, median [IQR]**	1.078	0.941-1.235
**AST, U/L, median [IQR]**	1.002	0.996-1.009
**ALT, U/L, median [IQR]**	0.999	0.989-1.008
**GGT, U/L, median [IQR]**	1.000	0.999-1.001
**Albumins, g/L, median [IQR]**	**0.865**	**0.803-0.932**
**Child Pugh score, points, median [IQR]**	1.363	0.937-1.981
**MELD, points, median [IQR]**	1.092	0.979-1.218
**FIB-4, points, median [IQR]**	**1.359**	**1.133-1.630**
**APRI, points, median [IQR]**	**1.474**	**1.008-2.154**
**ALBI, points, median [IQR]**	**3.934**	**1.915-8.084**
**Spleen length, mm, median [IQR]**	**1.039**	**1.018-1.060**
**LSM, kPa, median [IQR]**	**1.068**	**1.036-1.102**
**CAP, dB/m, median [IQR]**	0.998	0.992-1.004

Supplementary table 2. Univariate and multivariate logistic regression model assessing independent predictors associated with the SSM> 41.8 kPa (as the indicator of presence of critical varices) using old Fibroscan software (N=51). **Abbreviations:** ALBI: albumin-bilirubin score; ALT: alanine aminotransferase; AST: aspartate aminotransferase; APRI: AST to Platelet Ratio Index**;** BMI: body mass index; CAP: continuous attenuation parameter; dB/m: decibels per meter; CI: confidence interval; ECOG: Eastern Cooperative Oncology Group; FIB-4: Fibrosis-4; GGT: gamma glutamyl transferase; g/L: grams per liter; INR: international normalised ratio; IQR: interquartile range; kPa: kilopascal; LSM: liver stiffness measurement; M: median; MELD: model of end-stage liver disease; µmol/L: micromoles per liter; mmol/L: millimoles per liter; N: number; MASLD: Metabolic dysfunction-associated steatotic liver disease; PBC: primary biliary cholangitis; SSM: spleen stiffness measurement; U/L: units per liter

**Dependent variable: SSM>41.8 kPa**	**Odds ratio (univariate)**	**95% CI**
**Independent variables**		
**Age, years, median [IQR**]	1.013	0.968-1.061
**Age at time of diagnosis, years, median [IQR**]	1.007	0.950-1.067
**Male sex**	3.231	0.776-13.446
**BMI, kg/m ^{2}, median [IQR]**	1.118	0.956-1.308
**ECOG**<br>**0**<br>**1**<br>**2**	 Ref.<br>2.16<br>Not enough data	 <br>0.207-22.489
**Obesity (BMI>30 kg/m ^{2})**	3.117	0.745-13.037
**Arterial hypertension**	0.731	0.229-2.334
**Hyperlipidemia**	0.615	0.110-3.427
**Diabetes type II**	0.724	0.220-2.378
**Charlson Comorbidity Index**	1.031	0.822-1.293
**Etiology- Alcohol**	1.235	0.395-3.864
**Etiology- MASLD**	0.577	0.143-2.323
**Esophageal varices**	**29.467**	**6.214-139.732**
**Critical varices**	**13.722**	**1.625-115.9**
**Leukocytes, G/L, median [IQR]**	**0.671**	**0.488-0.924**
**Neutrophils, G/L, median [IQR]**	**0.623**	**0.422-0.920**
**Lymphocytes, G/L, median [IQR]**	**0.349**	**0.139-0.882**

**Neutrophile to lymphocyte ratio, median [IQR]**	0.963	0.645-1.436
**Hemoglobin, g/L median [IQR]**	1.023	0.993-1.053
**Platelets, G/L, median [IQR]**	0.977	0.965-0.989
**INR, median [IQR]**	2.239	0.265-18.942
**Bilirubin, µmol/L, median [IQR]**	**1.066**	**1.0001-1.137**
**Creatinine, µmol/L, median [IQR]**	**0.966**	**0.936-0.998**
**Sodium, U/L, median [IQR]**	1.204	0.961-1.509
**AST, U/L, median [IQR]**	1.008	0.989-1.027
**ALT, U/L, median [IQR]**	0.995	0.979-1.011
**GGT, U/L, median [IQR]**	1.0002	0.998-1.003
**Albumins, g/L, median [IQR]**	**0.900**	**0.816-0.993**
**Child Pugh score, points, median [IQR]**	1.792	0.883-3.638
**MELD, points, median [IQR]**	1.068	0.898-1.270
**FIB-4, points, median [IQR]**	**1.731**	**1.146-2.612**
**APRI, points, median [IQR]**	2.173	0.894-5.284
**ALBI, points, median [IQR]**	**3.539**	**1.237-10.124**
**Spleen length, mm, median [IQR]**	**1.043**	**1.009-1.077**
**LSM, kPa, median [IQR]**	**1.077**	**1.023-1.136**
**CAP, dB/m, median [IQR]**	0.997	0.989-1.005

Supplementary table 3. Univariate and multivariate logistic regression model assessing independent predictors associated with the SSM> 40.9 kPa (as the indicator of presence of critical varices) using new Fibroscan software (N=61). **Abbreviations:** ALBI: albumin-bilirubin score; ALT: alanine aminotransferase; AST: aspartate aminotransferase; APRI: AST to Platelet Ratio Index**;** BMI: body mass index; CAP: continuous attenuation parameter; dB/m: decibels per meter; CI: confidence interval; ECOG: Eastern Cooperative Oncology Group; FIB-4: Fibrosis-4; GGT: gamma glutamyl transferase; g/L: grams per liter; INR: international normalised ratio; IQR: interquartile range; kPa: kilopascal; LSM: liver stiffness measurement; M: median; MELD: model of end-stage liver disease; µmol/L: micromoles per liter; mmol/L: millimoles per liter; N: number; MASLD: Metabolic dysfunction-associated steatotic liver disease; PBC: primary biliary cholangitis; SSM: spleen stiffness measurement; U/L: units per liter

**Dependent variable: SSM>40.9 kPa**	**Odds ratio (univariate)**	**95% CI**
**Independent variables**		
**Age, years, median [IQR]**	0.999	0.952-1.049
**Age at time of diagnosis, years, median [IQR]**	1.003	0.955-1.054
**Male sex**	3.158	0.949-10.503
**BMI, kg/m ^{2}, median [IQR]**	1.027	0.927-1.138
**ECOG**<br>**0**<br>**1**<br>**2**	 Ref.<br>4.461<br>Not enough data	 <br>0.468-42.516
**Obesity (BMI>30 kg/m ^{2})**	1.467	0.480-4.477
**Arterial hypertension**	0.818	0.276-2.422
**Hyperlipidemia**	1.055	0.378-2.949
**Diabetes type II**	1.385	0.500-3.835
**Charlson Comorbidity Index**	**1.366**	**1.031-1.810**
**Etiology- Alcohol**	1.583	0.573-4.378
**Etiology- MASLD**	0.875	0.272-2.812
**Esophageal varices**	**8.186**	**2.568-26.093**
**Critical varices**	Insufficient data	
**Leukocytes, G/L, median [IQR]**	0.795	0.617-1.025
**Neutrophils, G/L, median [IQR]**	0.837	0.609-1.151

**Lymphocytes, G/L, median [IQR]**	**0.186**	**0.064-0.544**
**Neutrophile to lymphocyte ratio, median [IQR]**	1.372	0.922-2.042
**Hemoglobin, g/L median [IQR]**	**0.973**	**0.948-0.998**
**Platelets, G/L, median [IQR]**	**0.979**	**0.965-0.992**
**INR, median [IQR]**	**75.146**	**2.502-2256.494**
**Bilirubin, µmol/L, median [IQR]**	1.005	0.987-1.024
**Creatinine, µmol/L, median [IQR]**	1.019	0.991-1.048
**Sodium, U/L, median [IQR]**	0.969	0.809-1.163
**AST, U/L, median [IQR]**	1.002	0.996-1.008
**ALT, U/L, median [IQR]**	0.997	0.986-1.009
**GGT, U/L, median [IQR]**	1.0001	0.998-1.002
**Albumins, g/L, median [IQR]**	**0.827**	**0.736-0.928**
**Child Pugh score, points, median [IQR]**	1.249	0.809-1.927
**MELD, points, median [IQR]**	1.146	0.986-1.332
**FIB-4, points, median [IQR]**	**1.337**	**1.069-1.674**
**APRI, points, median [IQR]**	1.347	0.867-2.092
**ALBI, points, median [IQR]**	**4.659**	**1.683-12.894**
**Spleen length, mm, median [IQR]**	**1.046**	**1.015-1.078**
**LSM, kPa, median [IQR]**	**1.079**	**1.033-1.127**
**CAP, dB/m, median [IQR]**	0.996	0.986-1.007
